# MiR-183 Regulates ITGB1P Expression and Promotes Invasion of Endometrial Stromal Cells

**DOI:** 10.1155/2015/340218

**Published:** 2015-08-19

**Authors:** Jie Chen, Lin Gu, Jie Ni, Ping Hu, Kai Hu, Ying-Li Shi

**Affiliations:** ^1^State Key Laboratory of Reproductive Medicine, Department of Gynecology, Nanjing Maternity and Child Health Care Hospital Affiliated to Nanjing Medical University, 123 Tianfei Road, Nanjing, Jiangsu 210029, China; ^2^Shanghai Ji Ai Genetics & IVF Institute, Obstetrics & Gynecology Hospital, Fudan University, 588 Fangxie Road, Shanghai 200011, China

## Abstract

We applied in the previous study miRNA microarray screening analysis to identify several differentially expressed miRNAs, including miR-183 in normal, eutopic, and ectopic endometrium. Knockdown of miR-183 expression induced the invasiveness and inhibition of apoptosis in endometrial stromal cells. The current study aims to identify the miR-183 targets with relevance to cell functions in endometrial stromal cells, to verify the interaction of miR-183 with its target genes, and to confirm the role of miR-183 in the process of endometriosis. Using microarray analysis, we identified 27 differentially expressed genes (19 were upregulated and 8 downregulated), from which we selected 4 downregulated genes (ITGB1, AMIGO2, VAV3, and PSEN2) based on GO databases for functional analysis and significant pathway analysis. Western blotting analyses showed that integrin *β*1 (ITGB1), but not AMIGO2, was affected by miR-183 overexpression, whereas no protein expression of VAV3 and PSEN2 was detected. Luciferase reporter assay verified that ITGB1 is a target gene of miR-183. Moreover, we found that ITGB1 is overexpressed in the endometrium of endometriosis patients. Furthermore, overexpression of ITGB1 rescued the repressive effects of miR-183 on the invasiveness of endometrial stromal cells. These findings, together with the fact that ITGB1 is a critical factor for cell adhesion and invasiveness, suggest that miR-183 may be involved in the development of endometriosis by regulating ITGB1 in endometrial stromal cells.

## 1. Introduction

Affecting 6–10% of women at reproductive age, endometriosis is a common chronic gynecological disorder associated with infertility, severe pelvic pain, and menstrual disorders [1]. Despite intensive research efforts, the pathogenic mechanism of endometriosis remains poorly understood. So far, it is still a highly underdiagnosed disease, and the current surgical and hormonal treatment of endometriosis is not effective and recurrence is often observed.

MicroRNAs (miRNAs) are small, noncoding regulatory RNAs that regulate the stability and translation of mRNAs by inhibiting ribosome functions, deadenylating the poly(A) tail, and degrading target mRNA [2]. Aberrant miRNA expression has been linked to a variety of human diseases such as gynecological diseases, cancers, inflammatory diseases, and cardiovascular disorders [3].

A single microRNA molecule is able to modulate multiple target genes; thus it is involved in multiple cell functions including proliferation, invasiveness, and differentiation [4]. In 2007, Pan et al. detected deregulated expression of miRNAs in paired ectopic and eutopic endometrial tissues using microarray analysis. From then on, many studies have confirmed the aberrant expression patterns of miRNAs in ectopic as well as eutopic endometrial tissues from endometriotic patients. MiR-520, miR-199a, miR-23a, miR-23b, miR-145, miR-196, miR-10, miR-20a, let-7, miR-126, and miR-135 are among the most studied miRNAs in endometriosis. Adammek et al. reported that miR-145 inhibited endometriotic cell proliferation, invasiveness, and stemness by targeting pluripotency factors, cytoskeletal elements, and protease inhibitors [5]. MiR-196b was implicated in HOXA10 expression and in the proliferation of endometriotic stromal cells [6]. Loss of eutopic endometrial miR-451 expression is associated with decreased establishment of endometriosis in a mouse model [7]. These data landed strong support for an important role of miRNAs in the development of endometriosis through regulating cell proliferation, cell apoptosis, cell migration, cell invasiveness, and estrogenic signaling.

In a previous study, we identified several differentially expressed miRNAs among normal, eutopic, and ectopic endometrium. MiR-183 was found to be the most downregulated miRNA in both the ectopic and eutopic tissues compared to normal endometrium. Functional studies indicated that miR-183 may contribute to endometrial stromal cell apoptosis and impose a negative regulatory impact on cell invasiveness, but it has no effect on endometrial stromal cell proliferation. The study suggested that aberrant miR-183 expression may be involved in the development and progression of endometriosis [8].

In the present study, we aim to identify the functional targets of miR-183 using an overexpression model. We examine if miR-183 exerts its effects by directly binding to and inhibiting the ITGB1 promoter activity. In addition, we investigate if miR-183 could regulate stromal cell invasiveness. The findings from these experiments will help us to better understand the miR-183-mediated molecular mechanism in endometric stromal cells.

## 2. Materials and Methods

### 2.1. Tissue Acquisition and Cell Culture

All the eutopic and normal endometrial tissues were obtained at the Nanjing Maternity and Child Health Care Hospital Affiliated to Nanjing Medical University by uterine curettage from patients with or without endometriosis. None of the patients had received preoperative hormonal therapy, and all samples were histologically confirmed by pathologists. All samples were collected from the proliferative phase of the menstrual cycle. The phase of the menstrual cycle was determined based on histologic evaluation of the endometrium with the assistance of pathologists. The average age of the patients was 30.5 ± 5.2. Patients consented to tissue donation prior to surgery. The research project and consent form were approved by the hospital ethics committee. Each sample was divided into two parts: one part was snap-frozen and stored at −20°C and later used for mRNA and protein extraction; the other part was rinsed thoroughly with cold PBS and directly subjected to digestion and stromal cell isolation.

The endometrial stromal cells (ESC) from normal women without endometriosis were used as control. Tissue digestion and stromal cell isolation and culture were performed according to the method described in previous publication [9].

### 2.2. MiR-183-Lentivirus Construction and Transduction

The lentivirus gene transfer vector carrying precursor of hsa-miR-183 (Genbank accession number: MIMAT0000261) and encoding green fluorescence protein (GFP) were constructed by Genechem Co., Ltd., Shanghai, China, and confirmed by DNA sequencing. The primers of RNA were 5′-GAGGATCCCCGGGTACCAAGGGAGTGGGCAGGCTA and 5′-ATAAGCTTGATATCGTCCCTGCACCCTTGGAAGCA. The recombinant lentivirus for miR-183 overexpression (miR-183-lentivirus) and the control lentivirus (GFP-lentivirus) were prepared and titered to 5.0*E* + 8 TU/mL (transfection unit per mL).

The sequence of inhibitor of hsa-miR-183-5p was TATGGCACTGGTAGAATTCACT. The recombinant lentivirus of miR-183-5p inhibitor (In-miR-183 lentivirus) and the control lentivirus (GFP-lentivirus) were prepared and titered to 4.0*E* + 8 TU/mL (transfection unit per mL).

ESC from women without endometriosis were plated in 6-well plates (5 × 10^4^ cells/well) overnight. The lentiviruses were diluted in 0.2 mL complete medium containing polybrene (8 mg/mL) and added to the cells for 12 h of incubation at 37°C, followed by incubation in 0.3 mL of freshly prepared polybrene-DMEM for 24 h. The media were replaced with fresh DMEM and the cells were cultured for 3 days. The lentivirus transduction efficiency of ESC was determined by the detection of GFP signals under a fluorescence microscope at 72 h after transduction. The miR-183 expression in stably transduced ESC was measured by real-time PCR. The ESC transfected with miR-183-lentivirus, In-miR-183-lentivirus, and GFP-lentivirus were kept for further analysis.

### 2.3. RNA Extraction and Microarray

For the microarray analyses, groups were divided into the ESC with miR-183 overexpression and the control ones. Total RNA was extracted using TRIzol (Invitrogen) according to the manufacturer's instructions. Gene expression profiling was conducted using PrimeView Human Gene Expression Array (Affymetrix). The array contains 530,000 probes covering more than 36,000 transcripts and variants, which represent more than 20,000 genes mapped through RefSeq or via UniGene annotation. All subsequent technical procedures and quality controls were performed by Genechem Co., Ltd., Shanghai, China. The arrays were scanned using a GeneChip Scanner 3000 (Affymetrix, Inc., CA, USA). Raw data were extracted from the scanned images and analyzed using GeneSpring GX software version 11.5 (Agilent Technologies, CA, USA).

The data were normalized using the PLIER default protocol. Significant differentially expressed genes were analyzed using an unpaired *t*-test. Hierarchical cluster analysis was used to assess correlations among samples for each identified gene set with Euclidean distance and average linkage statistical methods.

### 2.4. Microarray Data Analyses

Differentially expressed genes were uploaded to the Ingenuity database (Ingenuity Systems, Redwood City, CA, USA) for pathway and functional analyses. Genes were further annotated and classified based on the Gene Ontology (GO) consortium annotations from the GO Bostaurus database using GOEAST (Gene Ontology Enrichment Analysis Software Toolkit).

### 2.5. Western Blotting

ESC proteins were extracted with RIPA buffer [(50 mM Tris-HCl, pH 8.0, 150 mM NaCl, 1% (v/v) NP-40, 0.5% (v/o) sodium deoxycholate, and 0.1% (w/o) sodium dodecyl sulphate (SDS)] supplemented with a protease inhibitor cocktail (Sigma). After incubating on ice for 30 min, cell debris was removed by centrifugation at 12,000 g for 10 min. Cell lysates were resolved in 12% SDS-PAGE electrophoresis and transferred to the PVDF membrane (Bio-Rad, Hercules, CA). Nonspecific binding was blocked by incubating the membranes in Tris-buffered saline containing 5% nonfat milk (TBST, 50 mmol/L Tris–HCl, 150 mmol/L NaCl, and 0.1% Tween-20) for 1 h at room temperature. Membranes were incubated overnight at 4°C with AMIGO2 antibody (1 : 400, RD), integrin *β*1 antibody (1 : 1000, Cell Signaling), Presenilin 2 antibody (1 : 500, ABCAM), and VAV3 antibody (1 : 500, ABCAM). After three washes with TBST, the membranes were incubated for 1 h at room temperature with the horseradish peroxidase-labeled secondary antibody. The signals were visualized with chemiluminescence and quantified with Quantity-One (Bio-Rad, America).

### 2.6. Luciferase Assay

The mutant construct of ITGB1 3′UTR was obtained by introducing the mutation into the 7 nucleotides (GUGCCAUU) of the seed region for miR-183. The mutant construct of AMIGO2 3′UTR was obtained by introducing two mutations into the 7 nucleotides (GUGCCAUA) (UGCCAUA) of the seed region for miR-183. The miR-183 target sequences in the coding region of ITGB1/AMIGO2 were amplified by PCR and cloned into GV143 that contained a firefly luciferase reporter gene. Wild-type ITBG1/AMIGO2 3′UTR or mutant ITBG1/AMIGO2 3′UTR and the empty 3′UTR vector were cotransfected into ESC. Cell transfection using Lipofectamine 2000 and normalization for transfection efficiency was performed according to the recommendation of manufacturer (Invitrogen). Luciferase activity was measured 24 h after transfection using the Dual-Luciferase Reporter Assay System (Promega Corp., Madison, WI).

### 2.7. Quantitative RT-PCR

Endometrial tissue was minced with a scalpel blade. Total RNA was isolated using TRIzol (Takara, Otsu, Shiga, Japan) and cDNA was synthesized using the SYBR Prime Script RT-PCR kit (Takara) on the ABI Prism 7500 Sequence Detection System according to manufacturer's instructions. The housekeeping gene *β*-actin was used for normalization. The primers used were 5′-TCGTCACGTTCCGGTTATTC (sense) and 5′-CTTTTACTTACGGTTTACCC (antisense) for ITGB1; 5′-CCTCGCCTTTGCCGATCCG (sense) and 5′-GCCGGAGCCGTTGTCGACG (antisense) for *β*-actin. The cycling conditions were 94°C for 1 min, 35 cycles of 94°C for 30 sec, 66°C for 30 sec, and 68°C for 3 min, followed by extension at 68°C for 3 min. Quantitative PCR was carried out using SYBR-Green JumpStart Taq ReadyMix (Sigma) and the 7300 Real-Time PCR Detection System (ABI). The results were analyzed using the comparative threshold cycle (CT) method.

### 2.8. Rescue Experiment

To further validate direct targeting of ITGB1 by miR-183, functional rescue experiment was performed by cotransfection with miR-183 mimic and plasmid constructs expressing ITGB1 in ESC using Lipofectamine 2000 (Invitrogen) as described above. The expression plasmid pcDNA-ITGB1, encoding human ITGB1, was purchased from Genechem. The presence of complete ITGB1 coding regions was confirmed by DNA sequencing.

### 2.9. Invasion (Matrigel) Chamber Assay

ESC (2.5 × 10^4^) were seeded on a transwell insert coated with extracellular matrix (ECM) (8 mm pore size, 24-well format; Corning Costar) in 2% FBS medium. Complete medium (10% FBS) was added to the lower chamber. To determine the amount of invasion, cells were incubated for 24 h and then removed from the upper chamber using a cotton swab. The invaded cells on the underside of the insert were fixed with methanol, stained with crystal violet for 2 min, and rinsed with phosphate buffered saline (PBS). The undersides of the membrane were photographed to compare the number of invaded cells per insert. The transmigrated cells were counted using a light microscope. Invasive cells were scored by counting 10 random high-power fields per filter.

### 2.10. Statistical Analyses

Data represents mean ± SEM from at least 3 independent experiments. Difference between experimental and control groups was determined by Student's *t*-test while one-way ANOVA analysis was employed to compare three or more groups. *P* < 0.05 was considered as statistical significance.

## 3. Results

### 3.1. Gene Expression Profiling following miR-183 Overexpression

**Figure 1:**
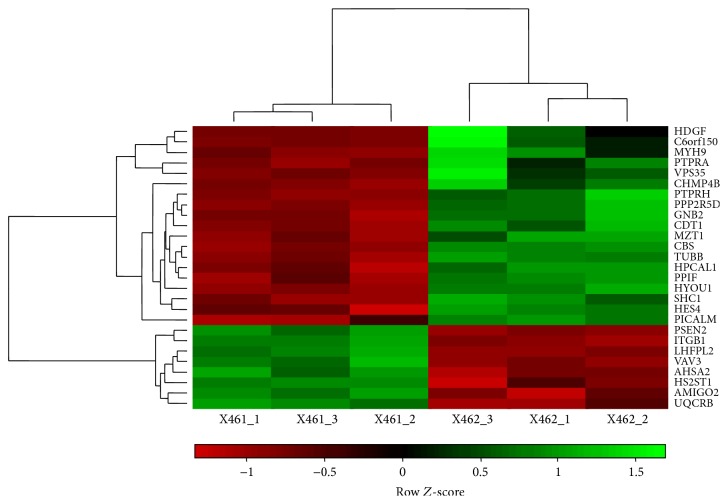
Hierarchical clustering of differentially expressed genes in miR-183-overexpressing endometrial stromal cells versus control cells. Gene expression profiling was conducted using PrimeView Human Gene Expression Array. Raw data were extracted from the scanned images and analyzed with GeneSpring GX software version 11.5. The data was normalized using the iterative PLIER default protocol. Differentially expressed genes were analyzed with the unpaired Student *t*-test. Statistically different genes (*P* < 0.05) with a greater than 1.5-fold increase or decrease were recorded. Red color indicates low expression; green color indicates relatively high expression.

In order to screen target genes in response to miR-183, we used microarrays representing more than 20,000 genes mapped through RefSeq or via UniGene annotation. We studied gene expression alterations (up- or downregulation) at 24 h after transfection. The changes of gene expression in miR-183-overexpressing endometrial stromal cells were analyzed. Differential expression was found in 27 genes at *P* value < 0.05 with folds of change ≥1.5. Of these, 19 were upregulated and 8 downregulated (ITGB1, AMIGO2, VAV3, PSEN2, LHFPL2, HS2ST1, AHSA2, and UQCRB). Results of hierarchical cluster analyses of these genes are shown in Figure 1 and supplementary 1 in Supplementary Material available online at http://dx.doi.org/10.1155/2015/340218.

### 3.2. Functional Analysis with GO Databases

By using the Gene Ontology (GO) database, we systematically extracted and analyzed the information of three GO categories, “biological process,” “molecular function,” and “cellular component.” It was revealed that the identified genes were involved in hemophilic cell adhesion (ITGB1, AMIGO2), cell-cell adhesion (ITGB1, AMIGO2), cell migration (ITGB1, MYH9), positive regulation of catalytic activity (PSEN2, SHC1), and proteolysis (MYH9, PSEN2) (Table 1).

### 3.3. Significant Pathway Analysis

Significant pathway analysis revealed that the gene expression alterations in endometrial stromal cells were involved in pathways of PTEN (ITGB1, SHC1), TFF (ITGB1, SHC1), ECM (ITGB1, SHC1), ERK (ITGB1, SHC1), integrin (ITGB1, SHC1), pathogenic* Escherichia coli* infection (ITGB1, TUBB), chemokine signaling pathway (SHC1, VAV3, and GNB2), focal adhesion (ITGB1, SHC1, and VAV3), regulation of cytoskeleton (ITGB1, VAV3, and MYH9), leukocyte transendothelial migration (ITGB1, VAV3), natural killer cell-mediated cytotoxicity (SHC1, VAV3), and Alzheimer's disease (UQCRB, PSEN2) (Table 2).

### 3.4. Confirmation of Microarray Data by Western Blotting

Because of the inhibitory property of miRNA on target genes, we chose from the list of 8 downregulated genes (ITGB1, AMIGO2, VAV3, PSEN2, LHFPL2, HS2ST1, AHSA2, and UQCRB) in miR-183-overexpressing cells. Biological function analysis using GO databases revealed that ITGB1 and AMIGO2 were involved in cell adhesion and/or cell migration. These two genes were selected for further study. PubMed reports showed VAV3 and PSEN2 were both involved in cell invasion [10, 11], and these two genes were included as study targets as well.

**Figure 2:**
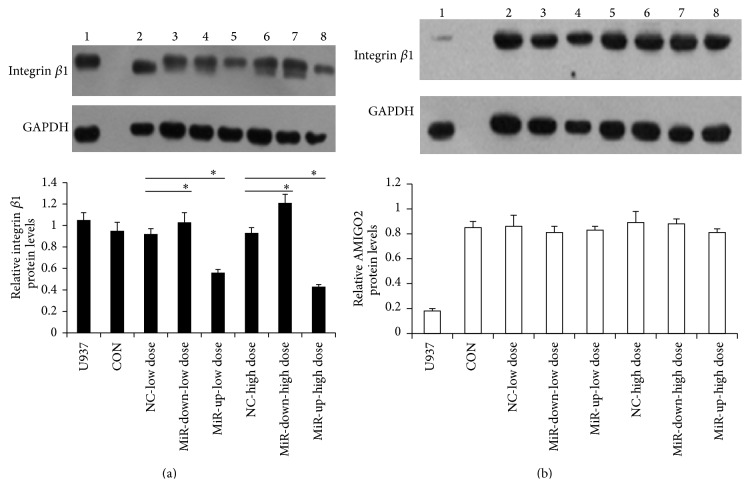
Confirmation of microarray data with Western blotting analyses. The endometrial stromal cells transfected with miR-183-lentivirus (low dose or high dose), In-miR-183-lentivirus (low dose or high dose), and GFP-lentivirus (NC, normal control) were used to examine the expression alterations of integrin *β*1 (a) and AMIGO2 (b). *β*-actin served as protein loading control. Compared with the control group, integrin *β*1 expression in the miR-183-overexpression group was significantly decreased, whereas it increased sharply in cells with low miR-183 expression. AMIGO2 was largely not regulated by miR-183. ^∗^ *P* < 0.05 when compared to the negative control. Error bars represent ± SEM.

Western blotting (Figure 2) was used to confirm their alterations on protein levels. Using the initial sample sets and the criteria of ≥1.5-fold of change, integrin *β*1 yielded consistent results by the two technologies. Compared to the control group, the levels of integrin *β*1 showed a significant decrease in the miR-183-overexpression group, whereas the miR-183-downexpressing group (*P* < 0.05) showed a sharp increase. Although endometrial stromal cells express AMIGO2 protein, the amount was largely unchanged in the miR-183 up- or downexpression groups, suggesting it was not regulated by miR-183. On the other hand, no protein expression of Presenilin 2 and VAV3 was detected in endometrial stromal cells (data not shown) and these two genes were not further studied.

### 3.5. MiR-183 Directly Targets ITBG1, but Not AMIGO2

**Figure 3:**
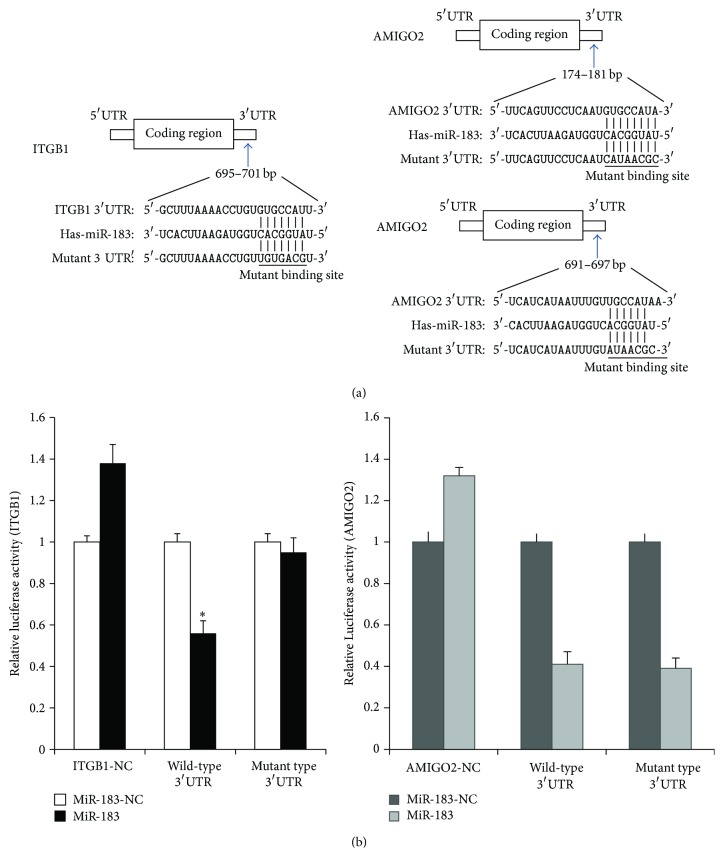
ITGB1, but not AMIGO2, is the direct target of miR-183. (a) The binding site of miR-183 in the 3′UTR of ITGB1 (top) and AMIGO2 (bottom) 3′UTR, along with the mutant construct in the predicted binding region. (b) MiR-183, but not AMIGO2, directly targets ITBG1. Endometrial stromal cells were cotransfected with wild-type reporter containing the ITBG1/AMIGO2 3′UTR or mutant ITBG1/AMIGO2 3′UTR plus miR-183 or negative control using Invitrogen Lipofectamine 2000 reagents. Luminescence was measured after 24 hours of transfection. Reporter plasmid expressing ITGB1 mRNA that contains sequences potentially targeted by miR-183 displayed a significantly decreased luciferase activity in cells transfected with miR-183 (*P* < 0.05), whereas luciferase activity of reporter plasmid containing mutant sequence of ITGB1 was not changed following forced expression of miR-183. ^∗^ *P* < 0.05 when compared to the negative control. Error bars represent ± SEM.

To investigate if ITBG1 and AMIGO2 are the direct targets of miR-183, we performed luciferase assay to determine whether miR-183 binds to the 3′UTR of ITBG1/AMIGO2 mRNA (Figure 3(a)). Our results showed that reporter plasmid containing the miR-183 targeting sequence of ITGB1 mRNA displayed a significantly decreased luciferase activity in cells transfected with miR-183 (*P* < 0.05), whereas luciferase activity of reporter plasmid containing mutant sequence of ITGB1 was not changed following forced expression of miR-183 (Figure 3(b)). We found no significant change when compared to the luciferase activity between cells transfected with wild-type or mutated AMIGO2 3′UTR (*P* > 0.05, Figure 3(b)). Thus, these data suggested that miR-183 negatively regulates the expression of ITBG1 by directly targeting their 3′UTR, whereas miR-183 does not directly target AMIGO2 3′UTR.

### 3.6. ITGB1 Is Overexpressed in Endometrium from Endometriosis Patients

**Figure 4:**
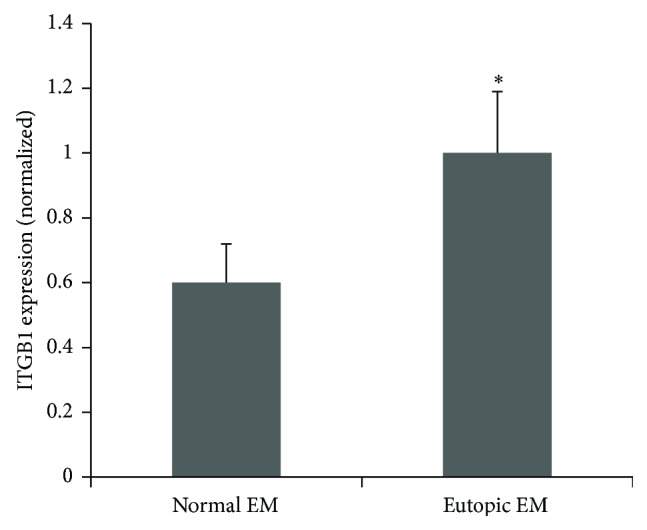
Validation of ITGB1 expression in eutopic endometrium with endometriosis (*n* = 19) versus normal group (*n* = 18) by qPCR. The results were analyzed using the comparative threshold cycle (CT) method. Gene expression data is presented as the fold of change relative to the levels of reference gene. ^∗^ *P* < 0.05 when compared to the normal group. Error bars represent ± SEM.

Real-time RT-PCR results indicated that ITGB was significantly increased in the eutopic endometrial tissues from patients with endometriosis (*n* = 19) compared with endometrium from normal women (*n* = 18) (*P* < 0.05) (Figure 4).

### 3.7. Overexpression of ITGB1 Rescued the Repressive Effects of miR-183 on Endometrial Stromal Cells

**Figure 5:**
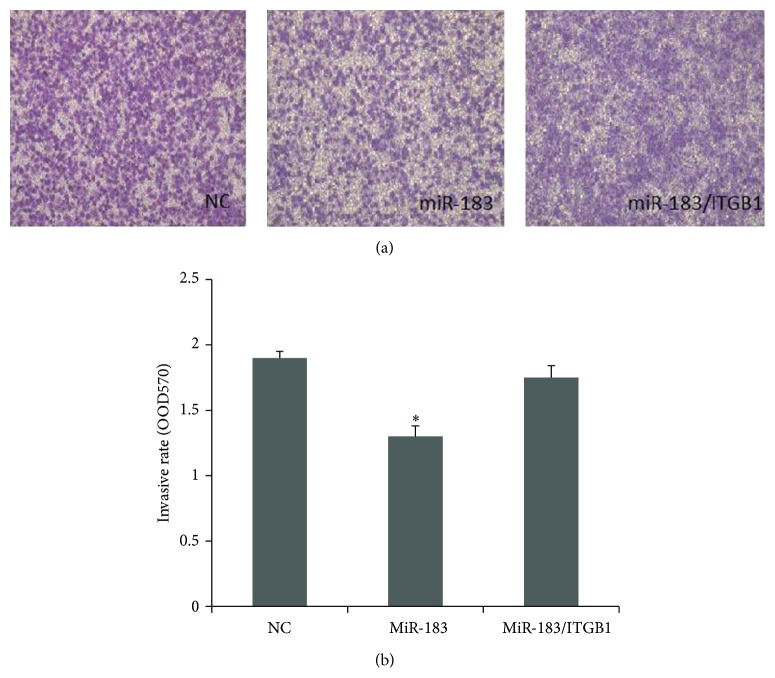
Overexpression of ITGB1 rescued the repressive effects of miR-183 on endometrial stromal cells, leading to elevated invasive abilities in transwell assays. Endometrial stromal cells were infected with miR-183-lentivirus, miR-183/ITGB1-lentivirus, and GFP-lentivirus in upper wells. After 24 hours, the number of cells that invaded through Matrigel was counted in at least 10 fields per well. (a) Representative photographs show that miR-183 inhibited the invasiveness of endometrial stromal cells, whereas ITGB1 partially rescued the repressive effects of miR-183. (b) Cell counting results indicate that ITGB1 overexpression rescued the repressive effects of miR-183. ^∗^ *P* < 0.05 when compared to the negative control. Error bars represent ± SEM.

To ascertain that miR-183 regulates the function of endometrial stromal cells through its interaction with ITGB1, a rescue experiment was performed. Overexpression of ITGB1 partially rescued the repressive effects of miR-183, leading to elevated invasion abilities in the cells (Figure 5). This data indicated that miR-183 targets ITGB1, which in turn led to negative regulation on the invasive activity of endometrial stromal cells.

## 4. Discussion

In the previous study, we performed miRNA microarray screening and identified several differentially expressed miRNAs in the normal, eutopic, and ectopic endometrium. Among these miRNA species, miR-183 was found to be downregulated in the ectopic and eutopic tissues. Functional analysis indicated that miR-183 promoted endometrial stromal cell apoptosis and had a negative regulatory impact on the invasive ability of cells, although it had no effect on cell proliferation [8]. These findings suggested that the downregulation of miR-183 expression might be involved in the pathogenesis of endometriosis. The molecular mechanisms, however, remain to be characterized.

MiR-183 is a member of a miRNA family (miR-183, miR-182, and miR-96) that clusters within a 2–4 kb region at chromosome 7q32. These miRNAs are known to regulate cell differentiation, apoptosis, motility, adhesion, and invasion [12]. Moreover, it has been reported that miR-183 is upregulated in colorectal cancer, prostate cancer, and hepatocellular carcinomas and downregulated in ovarian cancer, breast cancer stem cells, and osteosarcomas, pointing to varied and cell type-dependent function of miR-183. Several target genes of miR-183 including ITGB1, Taok1, Ezrin, EGR1, PDCD4, and LRP6 have been identified and validated [13–19].

To systematically investigate the potential mechanism by which miR-183 may contribute to the development of endometriosis, we performed microarray analysis to identify the target genes with relevance to cell functions in miR-183-overexpressing endometrial stromal cells. Eight genes were found to be downregulated in endometrial stromal cells with miR-183 overexpression. Among them, the GO databases, pathway analyses, and PubMed reports pointed to important association of 4 genes (ITGB1, AMIGO2, VAV3, and PSEN2) with cell adhesion, cell migration, and cell invasiveness. Western blotting results showed that endometrial stromal cell secreted integrin *β*1 and AMIGO2, but not VAV3 and Presenilin 2. The protein expression of integrin *β*1 appeared to be a direct regulation target of miR-183, which has been proved in human trabecular meshwork cells, human diploid fibroblasts, and hela cells [19]. AMIGO2 was not directly regulated by miR-183.

According to Sampson's theory, endometriosis is thought to be initiated via retrograde endometrium into peritoneal cavity during menstruation. However, while this phenomenon occurs in approximately 75–90% of women, far fewer suffer the disease, suggesting that additional factors must contribute to the process. Invasion to ectopic locations is the key step for the initiation of endometriosis, during which adhesion molecules play an important role. The integrin family includes glycoproteins that form dimeric structure and mediate cell attachment to the ECM components [20]. It has been reported that *β*1 integrin-depleted cells had reduced invasive capabilities, and *β*1 integrin signaling was involved in cancer cell invasion [21]. Integrin *β*1 is constitutively expressed in endometrial stromal cells and endometriotic lesions [22]. Increased integrin *β*1 protein expression in endometrial stromal cells was observed in endometriotic compared to normal endometrial tissues [23]. It was reported that downregulation of prostaglandin E2 receptors EP2 and EP4 led to inhibited adhesion of human endometriotic epithelial and stromal cells. Importantly, this inhibition could be mediated through suppression of integrin-mediated mechanisms [24]. This finding provided a strong evidence for the significance of integrins in the adhesion function of endometrial stromal cells.

AMIGO2 (ALI1, DEGA) was first reported to be preferentially expressed in the central nervous system. Sequence analyses revealed that the protein contains seven leucine-rich repeats, one IgC2-like loop, and a transmembrane domain and displays homology to Kek and Trk families [25–27]. In the gastric adenocarcinoma cell line, AMIGO2 was reported to be involved in cell adhesion, extracellular matrix, and basement membrane formation [28]. AMIGO2 was also implicated in oral carcinogenesis, by preventing the arrest of cell cycle progression at the G1 phase through inhibiting the expression of cyclin-dependent kinase inhibitors. However, little is known about the role of AMIGO2 in the development of endometriosis.

To investigate whether miR-183 directly targets ITGB1 and AMIGO2, dual luciferase reporter assays were carried out. We found that miR-183 negatively regulated the expression of ITBG1 by directly targeting its 3′UTR but might not directly regulate the expression of AMIGO2. We further detected differential expression of ITGB1 in endometrium with or without endometriosis by quantitative RT-PCR. The higher expression of integrin *β*1 in endometrium tissues in endometriosis was confirmed, which is consistent with the result for higher integrin *β*1 expression in ectopic stromal cells than normal endometrial stromal cells. Previous studies have shown that integrin *β*1 mediates cell adherence to extracellular matrix [29]. Indeed, the rescue experiment indicated that overexpression of ITGB1 blocked the repressive effects of miR-183 on endometrial stromal cell invasiveness, which provided a critical evidence supporting that the lower expression of miR-183 may promote ITGB1 expression and contribute to the development of endometriosis.

In conclusion, altered miR-183 expression may cause deregulation of its target gene ITBG1, affecting the cell adhesiveness and invasiveness of endometrial stromal cells, which in turn lead to pathogenesis of endometriosis. These findings revealed a novel pathologic role of miR-183 for endometriosis. It would be of great interest to investigate if these alterations may serve as biomarkers for the prediction and/or treatment of endometriosis. The regulation of miR-183 and the exact mechanism by which miR-183 regulates ITBG1 expression remain to be characterized.

## Supplementary Material

Suppl 1: The fold changes of differentially expressed genes in miR-183-overexpressing endometrial stromal cells comparing with the control cells. Gene expression profiling was conducted using PrimeView Human Gene Expression Array. The array contains 530,000 probes covering more than 36,000 transcripts and variants, which represent more than 20,000 genes mapped through RefSeq or via UniGene annotation. The arrays were scanned using a GeneChip Scanner 3000. Raw data were extracted from the scanned images and analyzed using GeneSpring GX software version 11.5. The data were normalized using the PLIER default protocol.

## Figures and Tables

**Table 1:** List of genes with fold of change ≥1.5 (*P* < 0.05) and their biological functions.

Gene	Gene set name	*P*
ITGB1, AMIGO2	Hemophilic cell adhesion	1.97*E* − 03
ITGB1, AMIGO2	Cell to cell adhesion	5.02*E* − 02
ITGB1, MYH9	Cell migration	6.10*E* − 02
PSEN2, SHC1	Positive regulation of catalytic activity	1.52*E* − 01
MYH9, PSEN2	Proteolysis	1.91*E* − 01

**Table 2:** List of genes with fold of change ≥1.5 (*P* < 0.05) and the pathways involved.

Gene	Gene set name	Pathways	*P*
ITGB1, SHC1	BIOCARTA_PTEN_PATHWAY	PTEN dependent cell cycle arrest and apoptosis	3.08*E* − 03

ITGB1, SHC1	BIOCARTA_TFF_PATHWAY	Trefoil factors initiate mucosal healing	4.19*E* − 03

ITGB1, SHC1	BIOCARTA_ECM_PATHWAY	Erk and PI-3 kinase are necessary for collagen binding in corneal epithelia	5.46*E* − 03

ITGB1, SHC1	BIOCARTA_ERK_PATHWAY	Erk1/Erk2 Mapk signaling pathway	7.39*E* − 03

ITGB1, SHC1	BIOCARTA_INTEGRIN_PATHWAY	Integrin signaling pathway	1.34*E* − 02

ITGB1, TUBB	KEGG_PATHOGENIC_ESCHERICHIA_COLI_INFECFECTION	Pathogenic *Escherichiacoli* infection	3.06*E* − 02

SHC1, VAV3, GNB2	KEGG_CHEMOKINE_SIGNALING_PATHWAY	Chemokine signaling pathway	5.75*E* − 02

ITGB1, SHC1, VAV3	KEGG_FOCAL_ADHESION	Focal adhesion	6.59*E* − 02

ITGB1, VAV3, MYH9	KEGG_REGULATION_OF_ACTIN_CYTOSKELETON	Regulation of actin cytoskeleton	7.82*E* − 02

ITGB1, VAV3	KEGG_LEUKOCYTE_TRANSENDOTHELIAL_MIGRATRATION	Leukocyte transendothelial migration	1.05*E* − 01
