# Supplementary material for: MiR-183 Regulates ITGB1P Expression and Promotes Invasion of Endometrial Stromal Cells
Source: Biomed Res Int. 2015 Aug 19;2015:340218. doi: 10.1155/2015/340218 (PMC4556833; doi:10.1155/2015/340218)
Supplement: Supplementary file 1 — Suppl 1: The fold changes of differentially expressed genes in miR-183-overexpressing endometrial stromal cells comparing with the control cells. Gene expression profiling was conducted using PrimeView Human Gene Expression Array. The array contains 530,000 probes covering more than 36,000 transcripts and variants, which represent more than 20,000 genes mapped through RefSeq or via UniGene annotation. The arrays were scanned using a GeneChip Scanner 3000. Raw data were extracted from the scanned images and analyzed using GeneSpring GX software version 11.5. The data were normalized using the PLIER default protocol. [file 340218.f1.pdf]

Suppl 1. Fold change of the differentially expressed genes in miR-183-overexpressing endometrial stromal cells *versus* control cells.

Gene Symbol	Fold change	Regulation([OE] vs [NC])
AHSA2	1.5218405	down
AMIGO2	1.5111315	down
HS2ST1	1.5038928	down
ITGB1	1.7719837	down
LHFPL2	1.5300908	down
PSEN2	1.6882111	down
UQCRB	1.6453439	down
VAV3	1.5934956	down
C6orf150	1.5548234	up
CBS	1.5090706	up
CDT1	1.6033745	up
CHMP4B	1.5956801	up
GNB2	1.529302	up
HDGF	1.5825031	up
HES4	1.5046576	up
HPCAL1	1.5164013	up
MYH9	1.5872067	up
MZT1	1.5085506	up
PICALM	1.7173792	up
PPIF	1.5092947	up
PPP2R5D	1.5321019	up
HYOU1	1.7329035	up
PTPRA	2.0059988	up
PTPRH	1.5089976	up
SHC1	1.5337036	up
TUBB	1.5212591	up
VPS35	1.8287842	up
